# Hepatocellular carcinoma among cirrhotics - Utility of screening and surveillance programs - Review article -

**Published:** 2014

**Authors:** R Opriță, IB Diaconescu, G Lupu, A Lupu, B Cristea, MR Bratu

**Affiliations:** * “Floreasca” Clinical Emergency Hospital, Bucharest; ** Department of Anatomy, “Carol Davila” University of Medicine and Pharmacy, Bucharest, Romania

**Keywords:** hepatocellular carcinoma, screening, AFP, prognosis

## Abstract

Screening of known cirrhotics for hepatocellular cancer (HCC) has long been a contentious topic. Studies to date have failed to conclusively prove or disprove the validity of α-fetoprotein (AFP) and hepatic ultrasound as screening mechanisms for HCC among cirrhotics. It is not clear whether these screening mechanisms provide any benefit in terms of reduced morbidity and mortality. Screening for HCC among cirrhotics by using AFP and/or imaging at every 6 months correlate with HCC diagnosis, thus portending better treatment options and an improved prognosis. Screening all the known cirrhotics for HCC may lead to decreased mortality.

## Introduction

Hepatocellular carcinoma (HCC) poses a considerable health risk worldwide; it is the fifth most common cancer in the world [**1**]. According to SEER (Surveillance, Epidemiology and End Results) data, HCC affects nearly 20 000 people each year in USA. However, this number is quickly increasing, making HCC more of a pressing concern. Both the incidence and mortality of HCC worldwide has doubled over the past 40 years, and it is expected to double again in the next 10 years [**2**,**3**]. Furthermore, HCC typically has an extremely severe prognosis by the time it is diagnosed. Most patients die within 1 year from diagnosis, and the 5 years survival is less than 10%. If there were screening methods, which could diagnose HCC earlier, many more effective therapeutic options would be available, thus improving the prognosis. For example, liver transplant for stage I and II of the disease in an appropriately selected population yields 5 years survival rates of 70–75% [**4**]. Clearly, the population who would benefit more out of the screening protocol must first be defined and determined. There is a 3–4% annual incidence of HCC among patients with compensated cirrhosis [**5**]. This high-risk population has been studied much more extensively in Asia than in North America.

## Materials and methods

The medical databases on the internet were searched for studies or book chapters analyzing the screening programs of HCC in cirrhotic patients. The most relevant ones were selected based on efficiently detecting HCC in risk patients and an assessment whether there were any differences of screening protocols was made.

## Results and discussions

**Studies**

In a large randomized controlled trial, conducted in China on 18 816 patients with chronic hepatitis B virus (HBV) infection or chronic hepatitis, the results of screening with α-fetoprotein (AFP) and ultrasound at every 6 months were compared for one group of patients with no screening for the other. Although only 58.2% from the screening group were diagnosed with HCC, they reported a lower mortality rate than the other group. Among those who developed HCC from the screening group, the 5 years survival rate was 46.4% and 0% in the other group. The mortality ratio was 0.63 [**6**]. However, in the Asian population, HBV plays a much more significant role, with up to 80% of HCC patients being positive for HBS antigen and 40% not having cirrhosis [**7**]. While both HBV and hepatitis C virus (HCV) carry an approximately 3–5% annual risk of HCC when associated with cirrhosis, it is known that HBV can progress to HCC in the absence of cirrhosis. Thus, ongoing HBV replication is a significant risk factor for HCC, although not necessarily HBV related cirrhosis [**5**]. The annual incidence of HCC arising from alcohol related cirrhosis is slightly less than 1–4% [**5**]. These differences within the cirrhotic population at risk for HCC raises the question if the screening protocol has different results in the Asian versus the American population.

Studies so far, regarding HCC screening among cirrhotics, have been conflicting and largely inconclusive, particularly those carried out in the American population. It is not clear whether the sensitivity and specificity of AFP and ultrasound are sufficient to lead to an earlier diagnosis of HCC and decreased mortality among cirrhotics [**6**,**7**].

Furthermore, AFP has long been a confounding factor in the question of HCC screening. While highly elevated AFP (>400 ng/ml) has a high specificity for the detection of HCC, lower indeterminate ranges may be indicative of either hepatitis flare and liver regeneration or HCC [**8**,**9**].

Also, approximately 10–20% of HCCs do not over express AFP, resulting in a sensitivity of only 64% for AFP values >400 ng/ml [**5**].

Routine liver ultrasound as a screening measure is also controversial because of its variability results between examiners. However, it is, nonetheless, the least expensive and invasive of the radiographic techniques. Sensitivity ranges from 11% to 99%, and specificity from 95% to 100% [**5**].

The risk for HCC in patients with advanced fibrosis has recently been highlighted in a study by Lok and colleagues [**11**] at 1%. Other studies have referenced it to be in the 2% to 5% range, including Bonis and colleagues [**11**] predictive model for the development of HCC, liver failure, or liver transplant for individual patients with chronic hepatitis C [**11**-**13**].

At this time, no marked regional differences in the incidence of HCC that are different or inconsistent with the regional prevalence of HCV infection seem to exist. The global differences in HCC incidence tend to follow the overall global incidence of viral hepatitis B or viral hepatitis C and the evolution to cirrhosis in HCV-positive patients. There are other risk factors for HCC that could increase the risk for liver cancer in certain regions that may be genotype dependent or related to exposure to aflatoxins, but these risks have most notably been seen in the hepatitis B setting, not hepatitis C [**12**,**13**].

**Surveillance and Screening**

Surveillance refers to the regular testing of an individual for a disease or problem - that is, the repeated application of screening tests. Screening is a term that should only be used to refer to the application of a diagnostic test in persons at risk. The standard screening and surveillance strategy for HCC in the setting of cirrhosis includes ultrasound examination, with the recommended surveillance interval of 6-12 months. Alpha-fetoprotein (AFP) measurement should be considered as a supplement to this screening and surveillance process, AFP alone should not be used for screening unless ultrasonography is not available [**12**]. This HCC screening/surveillance process has been found to be cost effective [**10**,**11**].

The AASLD [American Association for the Study of Liver Diseases] guidelines [**11**] on the management of HCC, highlights the importance of screening and surveillance. The guidelines go into extensive detail about surveillance by using AFP measurement and ultrasound examination at every 6-12 months but focus on ultrasound as the predominant surveillance method. Specific hepatitis B carrier groups are identified and recommended for screening/surveillance, including Asian men 40 years of age or older, Asian woman 50 years of age or older, all cirrhotic hepatitis B carriers, those with a family history of HCC, individuals from sub-Saharan Africa who are over 20 years of age and non-cirrhotic hepatitis B carriers with high serum HBV DNA levels [**14**]. The guidelines make a strong statement that patients with cirrhosis due to hepatitis C, cirrhosis due to alcohol, and cirrhosis due to other causes need to undergo screening and surveillance as well. As mentioned, this screening and surveillance process has been found to be cost effective [**3**]. Ultrasound examination has been reported to have a sensitivity ranging between 65% and 80% and a specificity > 90% when used as a screening test [**11**,**12**].The 6- to 12-months interval for surveillance has been proposed based on the doubling time of HCC at a median of 6 months (range, 1-19 months) [**5**].

Computed tomography (CT) scan and magnetic resonance imaging (MRI) do not have a specific role as first-line initial screening or surveillance tools for liver cancer. These tools are complementary to ultrasound when 1 of 2 clinical scenarios present: an increasing AFP level but a negative ultrasound, or an abdominal ultrasound that reports a suspected tumor. The other major setting for these other imaging modalities is when there is an abnormality found on ultrasound that needs to be confirmed by 1 or 2 imaging tests. On CT or MRI, one is expected to see a rapid vascular “blush” and then a rapid washout in the third and fourth phases of these vascular dynamic studies. These scans can serve, either alone or complementary, for the diagnosis of HCC and obviate the need for a biopsy of the tumor to prove its malignant origin [**6**]. CT scans are expensive and each CT scan has now a radiation risk that is equivalent to approximately 130 chest X-rays with a theoretical long-term risk for increased chances of post radiation malignancy. MRI scans appear to be safe, but nephrogenic sclerosing fibrosis may occur in patients who receive gadolinium. This has been predominantly seen in patients on dialysis, but informed consent for the use of gadolinium in patients with renal insufficiency needs to be completed [**8**].

AFP has been moved to a second-line test because of its poor sensitivity and specificity [**14**]. The specificity is low (at low AFP levels, and sensitivity is low when the AFP is > 100 ng/mL. Patients who are on the liver transplant waiting list almost universally have cirrhosis. Thus, these individuals will fall into the standard screening and surveillance process [**2**]. Newer biomarkers, such as des-gamma-carboxy prothrombin and AFP-L3, an isoform of AFP, are emerging as novel tools either for enhancing surveillance for HCC or for identifying those patients who have vascular invasion and who are at high risk for recurrent disease after different surgical or ablative procedures. All 3 of these biomarkers, AFP, AFP-L3 and des-gamma-carboxy prothrombin are FDA approved, and there are emerging data that may have utility in a number of clinical settings [**2**].

Finally, the annual cost of HCC in the United States was determined to be of about $454 million, with a per-patient cost of $32, 907, in terms of the considerable economic consequences [**1**,**3**]. Sarasin and colleagues [**14**] demonstrated the benefits of surveillance for HCC: the quality of the surveillance had a direct effect on HCC stage at time of detection, access to liver transplantation and survival.

There is no specific role for liver biopsy in screening or surveillance for HCC. However, liver biopsy has a role in those patients with indeterminate disease, with lesions typically between 1 and 2 cm, and, is warranted in the setting of cirrhosis without classic imaging criteria, or, the presence of a new liver mass in a patient without chronic liver disease or at low risk for HCC, especially if there is an atypical lesion or there is an absence of biomarkers. The use of liver biopsy to evaluate for HCC has now been relegated to a second- or third-line position. There are associated risks for tumor seeding and tumor tracking that range from 0% to about 12% [**12**].

There are major challenges in instituting effective screening and surveillance programs for HCC. One of the most important issues is the perception in the community that there is no treatment for HCC. Another major hurdle is the lack of disease surveillance that can lead to an increased risk for this liver malignancy. It is estimated that less than 50% of the individuals in the United States know that they are infected with HCV or hepatitis B virus.

There is also the issue of increased liver cancer risk in NASH (nonalcoholic steatohepatitis) induced cirrhosis, but this has not clearly been communicated to the hepatology, gastroenterology and primary care communities. It has also not been clearly communicated that HCC treatment includes potential cures, such as resection or ablation therapies or, more importantly, liver transplantation. Screening and surveillance has been deemed to be cost-effective and lifesaving, but this needs to be communicated to the general community as well. There are also technical issues with low-quality ultrasound studies due to the lack of training and also a lack of defined protocols. Effective screening for the disease states a subsequent institution of liver cancer surveillance techniques that are of high competence and, furthermore, recall programs that can be instituted through large healthcare systems or on an individual patient basis, which will all eventually help improve HCC outcomes.

## Conclusions

Incidence of HCC is rapidly increasing worldwide. Therefore, the early detection is critical in order to offer a curative therapy. It is unclear whether the screening of cirrhotics leads to the diagnosis of HCC at an earlier stage. Neither AFP measurement nor hepatic ultrasound showed to be efficient as a screening test. Cirrhotic patients at high risk of developing HCC should benefit from routine screening with AFP and/or hepatic ultrasound. Physicians should routinely screen known cirrhotic patients for HCC with AFP or hepatic ultrasound at every 6 months. Ideally, HCC will be more commonly detected in earlier stages, thus allowing improved treatment modalities and hopefully increased cure rate.
